# Reciprocal associations between affective decision-making and mental health in adolescence

**DOI:** 10.1007/s00787-022-02096-2

**Published:** 2022-10-17

**Authors:** Francesca Bentivegna, Eirini Flouri, Efstathios Papachristou

**Affiliations:** Department of Psychology and Human Development, UCL Institute of Education, University College London, 25 Woburn Square, London, WC1H 0AA UK

**Keywords:** Behaviour problems, Decision-making, Emotional problems, Gambling task, Millennium Cohort Study, Reward sensitivity

## Abstract

Poor affective decision-making has been shown to associate cross-sectionally with poor mental health in clinical populations. However, evidence from general population samples is scarce. Moreover, whether decision-making is prospectively linked to mental health in youth in the general population and whether such associations are reciprocal have yet to be examined. The present study examined bidirectional associations between various aspects of affective decision-making and emotional and behavioural problems at ages 11 and 14 years in 13,366 members of the Millennium Cohort Study. Decision-making (delay aversion, deliberation time, quality of decision-making, risk adjustment, risk-taking) and emotional (emotional symptoms, peer problems) and behavioural (conduct problems, hyperactivity/inattention) problems were measured using the Cambridge Gambling Task and the Strengths and Difficulties Questionnaire, respectively. Results of cross-lagged panel models adjusted for confounding revealed a negative reciprocal association between hyperactivity and quality of decision-making but also positive reciprocal associations between conduct problems and delay aversion, and between peer problems and deliberation time. Emotional problems and peer problems predicted a decrease in risk-taking, conduct problems predicted an increase in risk-taking, and hyperactivity predicted an increase in delay aversion and deliberation time. Furthermore, hyperactivity and conduct problems predicted less risk adjustment, and risk adjustment predicted fewer peer problems. The results suggest that behavioural problems are prospectively linked to greater risk-taking and lower risk adjustment in adolescence. Moreover, adolescents with behavioural problems tend to make poorer decisions and be more delay-averse, but also poorer quality of decision-making and increased delay aversion are associated with more behavioural problems over time.

**Supplementary Information:**

The online version contains supplementary material available at 10.1007/s00787-022-02096-2.

## Introduction

Affective decision-making (henceforth referred to as decision-making) is one of the main aspects of “hot” executive function whereby motivation (e.g. the potential for rewards) plays a fundamental role in deciding an outcome over another [1]. Therefore, decision-making is strongly linked to concepts such as reward sensitivity [2], in turn related to several mental health disorders in adulthood [3, 4]. Psychological research is also increasingly uncovering links between decision-making and mental health in children and adolescents [5–11]. For example, reward-hyposensitivity and diminished reward-seeking are directly relevant to the development of emotional problems [12]. Deficits in the activity of motivational circuitry during anticipation of rewards have been linked to behavioural problems, too [13]. As a strategic process of choice under risk, decision-making is commonly measured with gambling tasks [14, 15] such as the Cambridge Gambling Task (CGT) [16] and the Iowa Gambling Task (IGT) [17]. The CGT, for example, assesses six different aspects of decision-making, including delay aversion, deliberation time, quality of decision-making, risk adjustment, risk-taking, and overall proportion bet. Those participating in such tasks are aware of the risk when they place their bets, a situation mimicking real-world circumstances where a choice is more or less advantageous than another. Such tasks have the potential to identify specific aspects of decision-making which are implicated in specific mental illness symptoms and could thus serve as targets for interventions aiming to alleviate the burden associated with mental illness.

Decision-making measured using gambling tasks has been explored in adults with different mental health disorders [5–11], as well as in clinical youth populations. For example, children with attention-deficit/hyperactivity disorder (ADHD), typically characterised by impulsiveness and dysfunctional response inhibition [18], tend to show more risk-taking [19–24] and to be easily distracted by the prospect of an immediate reward [20, 21]. Moreover, a review about oppositional defiant disorder and conduct disorder highlighted that children and adolescents with these disorders show impaired functioning on the IGT [25]. As for internalising disorders, one study found that depressed adolescents are more sensitive to negative (versus rewarding) outcomes compared to healthy adolescents [26].

By contrast, much less is known about the link between decision-making and mental health in the general youth population. We know particularly little about the direction of such an association, with only very few longitudinal studies investigating reciprocal associations between decision-making (measured using gambling tasks) and internalising (emotional) or behavioural (externalising) problems in this population. One study examined the bidirectional relationships between peer problems and performance on the CGT in early and mid-adolescence [27]. That study showed that peer problems both reduced, and were reduced by, improved risk adjustment, i.e. the capacity to adjust risk-taking behaviour according to the likelihood of winning. However, both effects were weak and did not survive adjustment for confounders including family income, maternal education, ethnic background, stage of pubertal development, cognitive ability, baseline emotional and behavioural problems, and parental depressive symptoms. Another study found that decreased risk-taking predicted increased peer-reported anxiety symptoms, whilst higher levels of self- and teacher-reported anxiety predicted lower risk-taking [28]. As for other internalising problems, a recent study showed that increased levels of emotional problems and depressive symptoms were associated cross-sectionally and longitudinally, respectively, with a lower tendency to make risky decisions at the end of primary school [29], albeit both relationships were confounded by sex. Other cross-sectional research found a significant association between risk adjustment and emotional problems [30], but no association between depressive symptoms and risk-taking [31].

With respect to externalising problems, one longitudinal study found that more risk-taking predicted higher levels of conduct problems and hyperactivity [32], and that higher quality of decision-making predicted a decrease in both conduct problems and hyperactivity. Another study found that high risk-taking was positively related to aggression in both boys and girls, and oppositional defiant behaviour in girls only. However, this was only the case when these behavioural problems were peer-reported (compared to teacher-reported, where the associations were non-significant) [33]. Finally, one study found a negative relationship between reward sensitivity and delinquent behaviour, however, this was likely due to the mediating effect of problem-solving strategies [34].

Taken together, these findings suggest that decision-making and poor mental health are intertwined in general population samples of youth, particularly for behavioural problems, however, the evidence remains scant. They also highlight the need to disentangle the direction of the association between decision-making and mental health. Most studies to date typically assume that the impact of decision-making on mental health is unidirectional despite findings from clinical populations suggesting that it may be reciprocal [35]. The present study aimed to fill these gaps in the literature by making use of measures of performance on the CGT and various mental health outcomes at ages 11 and 14 years that were available in the UK’s largest and most recent birth cohort, the Millennium Cohort Study (MCS). We employed methodologically appropriate techniques to explore the potential reciprocity of the associations between decision-making and internalising problems (emotional and peer problems) and between decision-making and externalising problems (hyperactivity and conduct problems). Given that most of the evidence to date shows small effect sizes, it was hypothesised that we would find weak bidirectional associations. In addition, based on the available research in non-clinical samples, we expected the associations from increased risk-taking, worse risk adjustment and poorer quality of decision-making to later externalising problems to be stronger than those from earlier externalising problems to these aspects of decision-making.

## Methods

### Sample

The MCS is an ongoing UK cohort study, over-representing areas of socio-economic disadvantage in the four UK countries and areas of higher ethnic minority density in England, which has followed young people born between 2000 and 2002 [36]. The MCS includes information on 19,243 families, with the original sample comprising 18,818 cohort members. Seven sweeps of data have been completed, when participating children were aged 9 months, and 3, 5, 7, 11, 14 and 17 years, respectively. The productive number of families participating in the different sweeps was 18,522 (Sweep 1), 15,590 (Sweep 2), 15,246 (Sweep 3), 13,857 (Sweep 4), 13,287 (Sweep 5), 11,726 (Sweep 6), and 10,625 (Sweep 7), respectively. Measures of both decision-making and mental health were available when the participants were aged on average 11 (Sweep 5) and 14 (Sweep 6) years. Specifically, the mean age for Sweep 5 was 10.68(SD = 0.48; age range 10–12 years), and the mean age for Sweep 6 was 13.77 (SD = 0.45; age range 13–15 years). The analytic sample for this study was restricted to children with complete data on mental health (internalising and externalising symptoms) and the CGT in at least one time point (*N* = 13,366). This was done to increase the size of the analytic sample by using multiple imputation. In the case of twins and triplets, only the first-born child was included in the analyses to ensure independence of observations [37]. Interviews and self-report assessments were also completed by the cohort members’ parents/carers, who gave their informed consent prior to the beginning of the assessments (cohort members gave their assent at age 11 and consent at age 14). Ethical approval was obtained from NHS Multi-Centre Ethics Committees.

### Measures

*Decision-making* was measured with the CGT [16], a gambling task from the Cambridge Neuropsychological Test Automated Battery (CANTAB) [38] assessing various aspects of decision-making. The MCS interviewer followed a script whilst guiding the child through the task, and explanations regarding the structure of the assessment were provided prior to the beginning of the trials. The procedure took place at the cohort members’ homes and is as follows: children sit in front of a computer screen, are presented with ten boxes, either red or blue, and are told that a yellow token is hidden in one of the boxes. The task’s aim is to correctly guess in which box (red or blue) the token is hidden in five stages, each comprising several blocks of trials. In the first stage (decision-making stage), a binary decision has to be made with regard to whether the token is hidden in the red or the blue box. In the next stages (gambling stages), the participating child is given 100 points and is asked to bet a proportion of these points. The aim is to win as many points as they can. In correct bets, points are added and in incorrect ones, they are taken away. The current bet value is displayed in a circle in the centre of the screen and can incrementally increase or decrease depending on the task. The CGT produces six outcomes. Delay aversion, the difference in percentage bet in conditions where the bet value incrementally increases and in conditions where it incrementally decreases, reflects whether participants are prepared to wait to place a higher or lower bet; deliberation time, a measure of pre-motor processing and movement time, corresponds to the mean time taken (measured in milliseconds) to make a colour box response after the decision-making information has been presented; risk-taking, the mean proportion of points bet on trials where the most probable colour response is made, with more risk-taking indicating a higher sensitivity to reward/lower sensitivity to punishment; risk adjustment, the tendency to bet more points when the likelihood of correctly guessing where the token is hidden is high, i.e. when most boxes are either red or blue, compared to when that likelihood is low; quality of decision-making, the mean proportion of trials where the child bet on the most likely outcome, that is when the correct coloured box is chosen; finally, overall proportion bet, the mean proportion of points that are gambled across all trials. In the present study, overall proportion bet was excluded from the analyses because it correlated over 0.90 with risk-taking.

*Internalising and externalising problems* were measured with 20 items of the Strengths and Difficulties Questionnaire (SDQ) [39], a widely used questionnaire designed to screen for problems in four main domains, each measured with five items: emotional symptoms, conduct problems, hyperactivity/inattention, and peer relationship problems. Parents or carers were required to indicate whether each item about their children over the last six months was “not true” (0), “somewhat true” (1), or “certainly true” (2). Examples of these items are “often unhappy, down-hearted or tearful” for emotional symptoms; “often fights with other children or bullies them” for conduct problems; “constantly fidgeting or squirming” for hyperactivity; and “rather solitary, tends to play alone” for peer problems. Higher scores indicate more mental health difficulties. Cronbach’s alphas for the scales for Sweeps 5 and 6 were, respectively: 0.69 and 0.68 for emotional symptoms; 0.67 and 0.66 for conduct problems; 0.67 and 0.67 for hyperactivity; and 0.70 and 0.69 for peer problems.

*Key confounders* were selected based on literature suggesting significant associations with both internalising/externalising problems and decision-making [40–44]. These included child's gender, ethnicity, pubertal status, and cognitive ability, and maternal education. Both gender (male vs. female) and ethnicity (White vs. other) were treated as binary variables. A more granular distinction between ethnic groups was not feasible due to some of the ethnicities being under-represented in the sample. Pubertal status (some physical signs of puberty vs. no signs) at age 11 years was measured with the parent’s report at Sweep 5 of whether or not there was breast growth or menstruation or hair on body (for females), and voice change or facial hair or hair on body (for males). Maternal education (university degree or not) was measured using the mother’s National Vocational Qualification (NVQ) level. Finally, cognitive ability (IQ) in MCS was measured at age 5 using the British Ability Scales (BAS) of Naming Vocabulary, Pattern Construction and Picture Similarities, and the composite score derived by means of principal components analysis (PCA) was standardised to have a mean of 100 and a standard deviation of 15.

### Statistical analysis

All analyses were conducted using Stata version 17.0 [45]. First, differences between the analytic sample (*N* = 13,366) and cohort members excluded due to missing data (*N* = 5877) were examined. Because sample attrition over time was not random, this analysis allowed the estimation of the degree of sample selection bias, that is, the degree of generalisability of the results to the general population. Next, the correlations between decision-making and internalising and externalising problems at ages 11 and 14 years were assessed in the analytic sample. Finally, cross-lagged panel models were run to examine the relationships between the five decision-making outcomes (delay aversion, deliberation time, quality of decision-making, risk adjustment, and risk-taking) and the four scales of the SDQ (emotional symptoms, conduct problems, hyperactivity/inattention, and peer problems). Cross-lagged panel analysis is a statistical modelling technique which can be used to estimate simultaneously reciprocal associations between different measures across time (cross-lagged paths) as well as the stability of each measure across consecutive time points (autoregressive paths) [46, 47]. We ran nine independent cross-lagged panel models, one for each of the mental health and decision-making variables considered, both before (Model A) and after adjustments for confounding (Model B). To ensure that significant findings were not an artefact of inflated Type I error rate resulting from multiple testing, we also ran the analyses in a single analytic step which tested the bidirectional associations between all decision-making and mental health variables simultaneously. [Note: Here we present results from the independent models because some of the measures were highly correlated and, thus, collinear. The results in the single model remained materially unaltered and any differences are discussed in the Discussion section.] Missing data on covariates and outcome measures in the analytic sample were imputed using multiple imputation by chained equations (MICE) [48]. We generated 20 imputed datasets using linear, logistic and ordered regressions depending on the scale of measurement of the variables being imputed, and used Rubin’s combination rules to consolidate the obtained individual estimates into a single set of multiply imputed estimates. All models were run using study-specific attrition and stratification weights and cluster points to account for sample attrition and for the disproportionately stratified and clustered design of MCS [49]. Model fit was estimated using one fit index, the SRMR, derived from a model using complete data rather than the multiply imputed datasets as it is one of the few reliable fit indices that can be obtained when complex study designs are taken into account during the estimation process.

## Results

### Descriptive statistics

The analytic sample comprised 13,366 participants. Table S1 in the Supplementary materials illustrates the descriptive statistics for exposures, outcomes, and confounding variables, including frequencies, means and standard deviations, and percentage of missing data per variable (missingness ranged from 6.2 to 20.2% across all variables). Table S2 in the Supplementary materials shows results of the comparisons between the analytic and non-analytic samples. As expected, all SDQ scale mean scores in the analytic sample were significantly lower at both time-points compared to those in the non-analytic sample. Regarding decision-making, mean scores in risk adjustment were significantly higher in the analytic sample at both time-points. The mean score in quality of decision-making was significantly higher in the analytic sample only at age 11. The remaining CGT variables did not differ between the samples. Moreover, in the analytic sample, the mean cognitive ability score was significantly higher as were the proportions of females, those who were white and those with more educated mothers. Finally, no differences were found in the proportions of youth who showed signs of puberty between the two samples.

### Associations between decision-making and mental health at 11 and 14 years

Table S3 in the Supplementary materials summarises the Pearson’s correlation coefficients for the associations between the mental health and decision-making outcomes. With only few exceptions, the associations were statistically significant and in the expected direction, albeit weak (*r* < 0.30). Specifically, all four SDQ scales were negatively associated with quality of decision-making and risk adjustment at both time-points. Hyperactivity and peer problems showed positive correlations with delay aversion and deliberation time. However, whilst hyperactivity was associated with risk-taking at both ages 11 and 14 years, there was no association between peer problems at either age and risk-taking at age 14. All four SDQ symptom domains were also positively correlated with deliberation time at both time-points except for a non-significant relationship between deliberation time at age 11 and conduct problems at age 14. Finally, non-significant correlations were found between emotional symptoms at age 14 and risk-taking and delay aversion at age 11.

### Bidirectional relationships between decision-making and mental health at 11 and 14 years

**Fig. 1:**
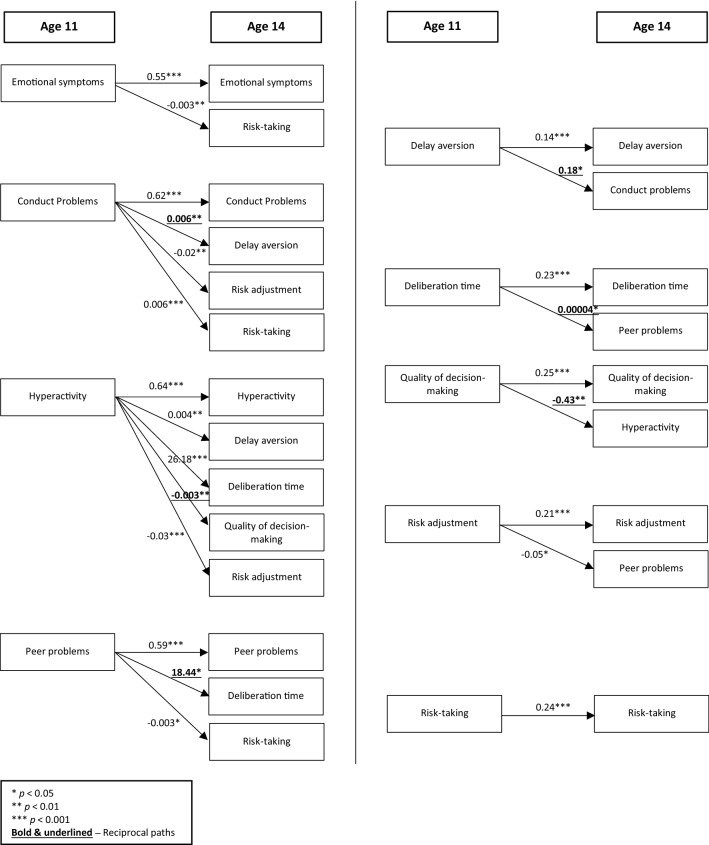
Significant paths (unstandardized regression coefficients) for SDQ at age 11 on the left-hand side, and for CGT at age 11 on the right-hand side, adjusted for child’s gender, ethnicity, pubertal status, and cognitive ability, and maternal education

Cross-lagged panel models were performed to explore potential reciprocal associations between decision-making and mental health at ages 11 and 14 years. Table S4 in the Supplementary materials and Table 1 summarise the results of the unadjusted (Model A) and adjusted (Model B) models, respectively. The SRMR for the model without MICE was less than 0.08, thus indicating “good” fit (SRMR = 0.042) [50]. Due to the small differences between the models, only the results from the adjusted models are reported here. The statistically significant paths found are illustrated in Fig. 1, whilst the standardised effect sizes are reported in Table S5 in the Supplementary materials.

**Table 1:** Results (unstandardized regression coefficients and their standard errors) of cross-lagged models examining the relationship between SDQ and CGT at age 11 with SDQ and CGT at age 14 (outcomes) adjusted for confounding

	SDQ age 14				CGT age 14				
	Emotional problems	Conduct problems	Hyperactivity	Peer problems	Delay aversion	Deliberation time	Quality of decision-making	Risk adjustment	Risk-taking
	b (SE)	b (SE)	b (SE)	b (SE)	b (SE)	b (SE)	b (SE)	b (SE)	b (SE)
**SDQ age 11**									
Emotional problems	0.55*** (0.01)	–	–	–	− 0.0003 (0.002)	− 1.45 (7.59)	0.0006 (0.001)	0.0006 (0.007)	− 0.003** (0.001)
Conduct problems	–	0.62*** (0.02)	–	–	0.006** (0.002)	2.66 (10.11)	− 0.001 (0.001)	− 0.02** (0.008)	0.006*** (0.001)
Hyperactivity	–	–	0.64*** (0.01)	–	0.004** (0.001)	26.18*** (6.44)	− 0.003** (0.001)	− 0.03*** (0.007)	0.001 (0.001)
Peer problems	–	–	–	0.59*** (0.01)	0.002 (0.002)	18.44* (9.10)†	− 0.0002 (0.001)	− 0.007 (0.008)	− 0.003* (0.001)
**CGT age 11**									
Delay aversion	0.03 (0.12)	0.18* (0.09)†	0.05 (0.10)	0.01 (0.09)	0.14*** (0.01)	–	–	–	–
Deliberation time	0.00002 (0.00002)	3.46e-06 (0.00001)	4.76e-06 (0.00002)	0.00004* (0.00002)	–	0.23*** (0.02)	–	–	–
Quality of decision-making	− 0.05 (0.16)	− 0.17 (0.12)	− 0.43** (0.14)	− 0.06 (0.12)	–	–	0.25*** (0.01)	–	–
Risk adjustment	− 0.02 (0.03)	− 0.02 (0.02)	− 0.03 (0.03)	− 0.05* (0.02)†	–	–	–	0.21*** (0.01)	–
Risk-taking	0.10 (0.18)	0.03 (0.14)	0.26 (0.16)	− 0.12 (0.12)	–	–	–	–	0.24*** (0.01)

Bold font indicates bidirectional relationships

Results refer to cross-lagged path analysis conducted for nine models separately. Model fit index (single analytic model): SRMR 0.042

Each model was adjusted for the respective autoregressive paths of SDQ and CGT measures and for the confounders, i.e. child’s gender, ethnicity, pubertal status, and cognitive ability, and maternal education

*SDQ* strengths and difficulties questionnaire, *CGT* Cambridge gambling task, *b* unstandardised coefficient, *SE* standard error

^*^p < 0.05; **p < 0.01; ***p < 0.001

^†^Associations that are not significant following multiple comparison adjustment

The results showed that the bidirectional associations between hyperactivity and quality of decision-making, and between peer problems and deliberation time found in Model A survived the adjustments for confounding; specifically, higher levels of hyperactivity at age 11 were associated with worse quality of decision-making at age 14 (*b* = − 0.003: *p* = 0.001), and increased quality of decision-making at age 11 was, in turn, associated with lower levels of hyperactivity at age 14 (*b* = − 0.43; *p* = 0.003). Moreover, higher levels of peer problems at age 11 were associated with more deliberation time at age 14 (*b* = 18.44; *p* = 0.043), whilst increased deliberation time at age 11 was associated with higher levels of peer problems at age 14 too (*b* = 0.00004; *p* = 0.028). A positive reciprocal relationship was also found between conduct problems and delay aversion with more conduct problems at age 11 being associated with more delay aversion at age 14 (*b* = 0.006; *p* = 0.008), whilst more delay aversion at age 11 was associated with more conduct problems at age 14 (*b* = 0.18; *p* = 0.048). In contrast to Model A, the reciprocal relationship of hyperactivity with risk-taking in Model B was no longer statistically significant. Regarding unidirectional associations, all the associations found in the unadjusted model remained significant in the adjusted one, with the only exception being the association between emotional problems at age 11 and risk adjustment at age 14. All the associations between internalising problems and decision-making were negative. Specifically, negative significant associations were found between peer problems at age 11 and risk-taking at age 14 (*b* = − 0.003; *p* = 0.030), between emotional symptoms at age 11 and risk-taking at age 14 (*b* = − 0.003; *p* = 0.008), and between risk adjustment at age 11 and peer problems at age 14 (*b* = − 0.05; *p* = 0.012). Instead, mostly positive associations were found for externalising problems. In particular, hyperactivity at age 11 was positively associated with delay aversion (*b* = 0.004; *p* = 0.002) and deliberation time (*b* = 26.18; *p* < 0.001) at age 14, whilst conduct problems at age 11 were positively associated with increased risk-taking at age 14 (*b* = 0.006; *p* < 0.001). As for negative associations, hyperactivity at age 11 (*b* = − 0.03; *p* < 0.001) and conduct problems at age 11 (*b* = − 0.02: *p* = 0.004) were both associated with a decrease in risk adjustment at age 14.

## Discussion

The aim of the study was to explore prospective and reciprocal associations between decision-making and psychological outcomes at ages 11 and 14 years in a large, general population birth cohort. The results suggest that, overall, poor decision-making in late childhood is associated with worse mental health in mid-adolescence, mainly externalising problems (hyperactivity and conduct problems). At the same time, these externalising problems in late childhood were predictive of worsening decision-making ability by mid-adolescence.

Three main results emerged. First, we found two reciprocal associations between externalising problems (conduct problems and hyperactivity) and decision-making, though we established more paths from earlier externalising problems to later decision-making than from earlier decision-making to later externalising problems. The unidirectional relationships from externalising problems to decision-making were as follows: a decrease in risk adjustment was predicted by both hyperactivity and conduct problems earlier, and an increase in risk-taking was predicted by earlier conduct problems. As for the reciprocal links, the reciprocal association between greater quality of decision-making and lower hyperactivity suggests that a child’s good-quality choices could help prevent the worsening of hyperactivity in adolescence, and that early interventions aimed at tackling hyperactivity and inattention symptoms in childhood could improve adolescents’ ability to make good-quality choices. The reciprocal association between conduct problems and delay aversion also makes sense when viewed in the context of previous literature suggesting that poor decision-making is linked with behavioural problems in clinical samples of children [21]. In our study, externalising problems were associated with CGT subscales more often than internalising problems (emotional symptoms and peer problems) were, thus further suggesting that affective decision-making and behavioural problems are closely linked in the general adolescent population. Given the role of externalising problems in later adverse outcomes across life domains [51], policy-makers should focus on their reduction and prevention not only for improving decision-making but a range of other outcomes too.

With respect to hyperactivity in particular, which was consistently associated with all the CGT subscales apart from risk-taking, our results are in line with previous findings on clinical samples which have demonstrated that children with ADHD are more likely to display impaired decision-making ability compared to controls [19–23]. To our knowledge, this is the first study looking at the reciprocal relationship between hyperactivity and decision-making in a general population youth sample. Therefore, this study provides novel information to the existing literature on a relationship that has been mainly explored in adults or amongst clinical samples of youth [52]. Surprisingly, we did not find an association between hyperactivity and risk-taking following adjustment for confounding, unlike much existing research [19–23]. One explanation for this is that the studies finding associations used clinical samples, i.e. children and/or adolescents diagnosed with ADHD. It might be that an association between hyperactivity and risk-taking is not visible in individuals with sub-clinical hyperactivity. At the same time, another study using data from the general youth population found that risk-taking was related to hyperactivity at age 11 [32]. One reason for this could be that this association disappears later in adolescence. Future studies should try to replicate these findings using samples drawn from the general population and at different developmental stages.

Second, we found a bidirectional relationship between peer problems and deliberation time, meaning that more time spent making a decision in childhood was associated with more peer problems in adolescence, and experiencing more peer problems in childhood was associated with taking longer to make decisions in adolescence. Peer problems in childhood were also associated with less risk-taking in adolescence, whereas greater risk adjustment in childhood was associated with fewer peer problems in adolescence. Our findings are in line with a previous study that found similar associations between bullying involvement in this age group and deliberation time, risk-taking, and risk adjustment [27].

Finally, emotional symptoms in childhood predicted a decrease, rather than an increase, in risk-taking in adolescence. This result corroborates evidence from samples of adults of a significant association between depressive symptoms and reduced sensitivity to rewards as measured using gambling tasks [9]. A similar association was found in a study that investigated the reciprocal association between anxiety levels and risk-taking, where there was evidence of a reciprocal relationship [28]. Instead, in our study, a relationship between early risk-taking and later emotional symptoms was not present. However, a previous study found that the longitudinal association between risk-taking and emotional and depressive symptoms disappeared after adjustment for confounding [29]. More research is needed to understand the direction of the relationship between emotional problems and decision-making.

This study had a number of strengths. It made use of a large sample which provided enough power to detect even small effect sizes. Moreover, the longitudinal study design and the availability of prospectively assessed measures enabled us to disentangle the direction of the relationships between decision-making and mental health. Nonetheless, our study had limitations too, and they should be taken into consideration when interpreting the results. First, some of the effect sizes we found were small; however, small effect sizes can still be relevant at the population level. Of note, three of the paths that were statistically significant at the 0.05 level of significance only (risk adjustment at 11 with emotional problems at 14 years, deliberation time at 11 with peer problems at 14 years, and peer problems at 11 with risk-taking at 14 years), were not found to be statistically significant in an additional analytic model which we ran in an exploratory manner and that examined all possible bidirectional associations simultaneously. It is therefore possible that the type I error rate of our study was non-negligible owing to the high number of tests that we ran. Nonetheless, the rest of the results remained materially unchanged suggesting that our findings are robust. Additionally, the MCS, like most population-based cohort studies, is subject to attrition and non-response. However, attrition and non-response were accounted for using study-specific weights in all analyses. Second, the Cronbach’s alphas for the SDQ were between 0.66 and 0.70. Thus, the reliability of some of the SDQ subscales in this study is questionable. Cronbach’s alpha measures the internal consistency (or reliability) of a scale, and a cut-off of ≥ 0.70 is generally deemed to be acceptable [53, 54]. Next, our sample bias analysis showed that cohort members in the analytic sample scored lower on the SDQ subscales and showed better decision-making ability compared to those in the non-analytic sample at both time points. As a result, the external validity of the study is somewhat compromised and some of the findings might not apply to populations at-risk for mental health difficulties. In addition, in MCS, decision-making on the CGT was first measured at age 11 years, thus precluding the possibility to investigate associations with mental health at earlier ages. Because mental health symptoms can start emerging already in early childhood [55], future studies should explore relationships between mental health and decision-making in younger samples. Also, it should be noted that the CGT has not been normed in samples of children. However, previous studies have used it with both high-risk [12] and clinical [56, 57] youth populations. Furthermore, despite the advantages of using cross-lagged path models, such models can only establish links that are still associative in nature and thus causal associations between the repeatedly assessed measures cannot be inferred [58]. Finally, because hyperactivity and conduct problems (themselves closely inter-related [59]) were both predictive of later delay aversion, it is possible that one of these externalising problems explains the association of delay aversion with the other. It would be informative if future studies investigated this possibility.

In conclusion, our study contributed to the current literature examining the relationships between decision-making and mental health in adolescence by focussing on potential reciprocal relationships between the two. We found a negative reciprocal association between hyperactivity and quality of decision-making, and positive reciprocal associations between peer problems and deliberation time, and between conduct problems and delay aversion. Unidirectional longitudinal associations were found between all the SDQ domains at age 11 and a least one of the decision-making domains examined at age 14, whilst only one unidirectional association was found between CGT (risk adjustment) at age 11 and SDQ (peer problems) at age 14. Taken together, the results of this study suggest a tendency for mental health problems to impair later decision-making. Moreover, it seems that behavioural rather than emotional problems are associated with poor decision-making, as shown in their links with particularly greater risk-taking and lower risk adjustment. Perhaps the most important conclusion from this study is that it appears that adolescents with behavioural problems tend to make poorer decisions and be more delay-averse, and that poorer quality of decision-making and increased delay aversion are associated with more behavioural problems over time.

### Supplementary Information

Below is the link to the electronic supplementary material.

Supplementary file1 (DOCX 54 KB)

## Data Availability

Data are publicly available via the UK Data Service.

## Code availability

Available upon request from the corresponding author.
