# Supplementary material for: Reciprocal associations between affective decision-making and mental health in adolescence
Source: Eur Child Adolesc Psychiatry. 2022 Oct 17;32(12):2513–22. doi: 10.1007/s00787-022-02096-2 (PMC10682223; doi:10.1007/s00787-022-02096-2)
Supplement: Supplementary file 1 — Supplementary file1 (DOCX 54 KB) [file 787_2022_2096_MOESM1_ESM.docx]

**Supplementary materials**

**Reciprocal associations between affective decision-making and mental health in adolescence**

Francesca Bentivegna^1^, Eirini Flouri^1^, Efstathios Papachristou^1^

^1^ Department of Psychology and Human Development, UCL Institute of Education, University College London, London, UK

Table of Contents

[Table S1 – Descriptive analyses for exposure, outcomes, and confounding variables, including missingness 2](#_Toc108617485)

[Table S2 – Unweighted sample bias analysis between analytic and non-analytic samples (continuous and categorical variables) 4](#_Toc108617486)

[Table S3 – Pairwise correlation analyses between SDQ and CGT at ages 11 and 14 years (unweighted data) 6](#_Toc108617487)

[Table S4 – Model A: Results of unadjusted cross-lagged models examining the relationship between SDQ and CGT at age 11 with SDQ and CGT at age 14 (outcomes) 7](#_Toc108617488)

[Table S5 – Model B: Results (standardised regression coefficients and their standard errors) of adjusted cross-lagged models examining the relationship between SDQ and CGT at age 11 with SDQ and CGT at age 14 (outcomes) 8](#_Toc108617489)

[MeSH Terms used in literature search in PubMed 9](#_Toc108617490)

Table S1 – Descriptive analyses for exposure, outcomes, and confounding variables, including missingness

| **Exposure and outcomes** | **Analytic sample** | |
| --- | --- | --- |
|  | **Sample**  **M (SD)** | **Missingness**  **N (%) – out of 13,336** |
| **Age 11** |  |  |
| **Child’s SDQ scores** |  |  |
| Emotional problems | 1.86 (1.99) | 830 (6.2) |
| Conduct problems | 1.37 (1.57) | 828 (6.2) |
| Hyperactivity | 3.10 (2.46) | 856 (6.4) |
| Peer problems | 1.36 (1.67) | 825 (6.2) |
| **Child’s CGT scores** |  |  |
| Delay aversion | 0.29 (0.25) | 1,046 (7.8) |
| Deliberation time | 3331.92 (1335.14) | 984 (7.4) |
| Quality of decision making | 0.80 (0.17) | 984 (7.4) |
| Risk adjustment | 0.65 (1.03) | 985 (7.4) |
| Risk-taking | 0.53 (0.17) | 985 (7.4) |
|  |  |  |
| **Age 14** |  |  |
| **Child’s SDQ scores** |  |  |
| Emotional problems | 2.04 (2.13) | 2,209 (16.5) |
| Conduct problems | 1.40 (1.62) | 2,207 (16.5) |
| Hyperactivity | 2.97 (2.39) | 2,214 (16.6) |
| Peer problems | 1.72 (1.80) | 2,204 (16.5) |
| **Child’s CGT scores** |  |  |
| Delay aversion | 0.27 (0.22) | 2,703 (20.2) |
| Deliberation time | 2338.12 (946.73) | 2,698 (20.2) |
| Quality of decision making | 0.88 (0.13) | 2,698 (20.2) |
| Risk adjustment | 0.99 (0.98) | 2,699 (20.2) |
| Risk-taking | 0.52 (0.15) | 2,699 (20.2) |
|  |  |  |
| **Covariates** |  |  |
| **Child’s cognitive ability^a^** | 100.72 (14.78) | 1,085 (8.1) |
|  |  |  |
|  | **N (%)** | **N (%)** |
| **Covariates** |  |  |
| **Child’s gender** |  | 0 (0) |
| Male | 6,730 (50.3) |  |
| Female | 6,636 (49.7) |  |
| **Child’s ethnicity** |  | 0 (0) |
| White | 11,086 (82.9) |  |
| Other than white | 2,280 (17.1) |  |
| **Child’s pubertal status^b^** |  | 943 (7.1) |
| Not yet started to show | 4,799 (38.6) |  |
| Started to show | 7,624 (61.4) |  |
| **Maternal education^b^** |  | 2,205 (16.5) |
| No higher education | 6,618 (59.3) |  |
| Higher education | 4,543 (40.7) |  |

Notes:

^a^ measured at age 5.

^b^ measured at age 11.

N total number of cohort members. M mean. SD standard deviation. *%* percentages with number of non-missing values as the denominator. SDQ Strengths and Difficulties Questionnaire. CGT Cambridge Gambling Task. F one-way ANOVA. *X^2^* Pearson’s Chi-Square.

Table S2 – Unweighted sample bias analysis between analytic and non-analytic samples (continuous and categorical variables)

|  | **Analytic sample** | | **Non-analytic sample** | | **Test of difference** |
| --- | --- | --- | --- | --- | --- |
|  | **N** | **Mean (SD)** | **N** | **Mean (SD)** | **F** |
| **Age 11** |  |  |  |  |  |
| **Child’s SDQ scores** |  |  |  |  |  |
| Emotional problems | 12,536 | 1.86 (1.99) | 259 | 2.49 (2.43) | 25.50*** |
| Conduct problems | 12,538 | 1.37 (1.57) | 260 | 2.13 (1.80) | 58.94*** |
| Hyperactivity | 12,510 | 3.10 (2.46) | 261 | 4.48 (3.05) | 79.52*** |
| Peer problems | 12,541 | 1.36 (1.67) | 261 | 2.37 (2.37) | 91.77*** |
| **Child’s CGT scores** |  |  |  |  |  |
| Delay aversion | 12,320 | 0.29 (0.25) | 128 | 0.30 (0.27) | 0.18 |
| Deliberation time | 12,382 | 3331.92 (1335.14) | 132 | 3389.14 (2264.58) | 0.24 |
| Quality of decision making | 12,382 | 0.80 (0.17) | 132 | 0.76 (0.19) | 9.64** |
| Risk adjustment | 12,381 | 0.65 (1.03) | 132 | 0.43 (1.14) | 5.74* |
| Risk-taking | 12,381 | 0.53 (0.17) | 132 | 0.55 (0.17) | 1.78 |
|  |  |  |  |  |  |
| **Age 14** |  |  |  |  |  |
| **Child’s SDQ scores** |  |  |  |  |  |
| Emotional problems | 11,157 | 2.04 (2.13) | 172 | 2.92 (2.59) | 29.35*** |
| Conduct problems | 11,159 | 1.40 (1.62) | 172 | 2.38 (2.12) | 61.94*** |
| Hyperactivity | 11,152 | 2.97 (2.39) | 172 | 4.59 (2.97) | 78.02*** |
| Peer problems | 11,162 | 1.72 (1.80) | 172 | 3 (2.30) | 84.28*** |
| **Child’s CGT scores** |  |  |  |  |  |
| Delay aversion | 10,663 | 0.27 (0.22) | 42 | 0.26 (0.32) | 0.18 |
| Deliberation time | 10,668 | 2338.12 (946.73) | 42 | 2499.83 (910.98) | 1.22 |
| Quality of decision making | 10,668 | 0.88 (0.13) | 42 | 0.87 (0.14) | 0.41 |
| Risk adjustment | 10,667 | 0.99 (0.98) | 42 | 0.61 (0.74) | 6.39** |
| Risk-taking | 10.667 | 0.52 (0.15) | 42 | 0.54 (0.17) | 1.20 |
|  |  |  |  |  |  |
| **Covariates** |  |  |  |  |  |
| **Child’s cognitive ability^a^** | 12,281 | 100.72 (14.78) | 2,582 | 96.60 (15.59) | 162.51*** |
|  |  |  |  |  |  |
|  | ***N*** | ***%*** | ***N*** | ***%*** | ***X^2^*** |
| **Covariates** |  |  |  |  |  |
| **Child’s gender** |  |  |  |  |  |
| Male | 6,730 | 50.3 | 3,159 | 53.9 |  |
| Female | 6,636 | 49.7 | 2,706 | 46.1 | 20.11*** |
| **Child’s ethnicity** |  |  |  |  |  |
| White | 11,086 | 82.9 | 4,646 | 79.1 |  |
| Other than white | 2,280 | 17.1 | 1,231 | 20.9 | 41.37*** |
| **Child’s pubertal status^b^** |  |  |  |  |  |
| Not yet started to show | 4,799 | 38.6 | 107 | 42.0 |  |
| Started to show | 7,624 | 61.4 | 148 | 58.0 | 1.17 |
| **Maternal education^b^** |  |  |  |  |  |
| No higher education | 6,618 | 59.3 | 209 | 70.9 |  |
| Higher education | 4,543 | 40.7 | 148 | 29.1 | 15.93*** |

Notes:

^a^ measured at age 5.

^b^ measured at age 11.

* p < 0.05; ** p < 0.01; *** p < 0.001

N total number of cohort members. M mean. SD standard deviation. *%* percentages with number of non-missing values as the denominator. SDQ Strengths and Difficulties Questionnaire. CGT Cambridge Gambling Task. F one-way ANOVA. *X^2^* Pearson’s Chi-Square.

Table S3 – Pairwise correlation analyses between SDQ and CGT at ages 11 and 14 years (unweighted data)

|  | 1. | 2. | 3. | 4. | 5. | 6. | 7. | 8. | 9. | 10. | 11. | 12. | 13. | 14. | 15. | 16. | 17. | 18. |
| --- | --- | --- | --- | --- | --- | --- | --- | --- | --- | --- | --- | --- | --- | --- | --- | --- | --- | --- |
| Age 11 |  |  |  |  |  |  |  |  |  |  |  |  |  |  |  |  |  |  |
| 1. Emotional problems | 1.00 |  |  |  |  |  |  |  |  |  |  |  |  |  |  |  |  |  |
| 2. Conduct problems | 0.38*** | 1.00 |  |  |  |  |  |  |  |  |  |  |  |  |  |  |  |  |
| 3. Hyperactivity | 0.38*** | 0.55*** | 1.00 |  |  |  |  |  |  |  |  |  |  |  |  |  |  |  |
| 4. Peer problems | 0.47*** | 0.37*** | 0.37*** | 1.00 |  |  |  |  |  |  |  |  |  |  |  |  |  |  |
| 5. Delay aversion | 0.02* | 0.07*** | 0.11*** | 0.05*** | 1.00 |  |  |  |  |  |  |  |  |  |  |  |  |  |
| 6. Deliberation time | 0.05*** | 0.03** | 0.05*** | 0.06*** | -0.15*** | 1.00 |  |  |  |  |  |  |  |  |  |  |  |  |
| 7. Quality of decision-making | -0.08*** | -0.08*** | -0.11*** | -0.07*** | -0.06*** | -0.20*** | 1.00 |  |  |  |  |  |  |  |  |  |  |  |
| 8. Risk adjustment | -0.06*** | -0.10*** | -0.12*** | -0.08*** | -0.16*** | -0.04*** | 0.28*** | 1.00 |  |  |  |  |  |  |  |  |  |  |
| 9. Risk-taking | -0.0003 | 0.10*** | 0.14*** | 0.05*** | 0.18*** | -0.06*** | 0.10*** | -0.18*** | 1.00 |  |  |  |  |  |  |  |  |  |
| Age 14 |  |  |  |  |  |  |  |  |  |  |  |  |  |  |  |  |  |  |
| 10. Emotional problems | 0.55*** | 0.29*** | 0.28*** | 0.33*** | 0.009 | 0.05*** | -0.07*** | -0.07*** | -0.02 | 1.00 |  |  |  |  |  |  |  |  |
| 11. Conduct problems | 0.27*** | 0.61*** | 0.43*** | 0.28*** | 0.07*** | 0.02 | -0.08*** | -0.10*** | 0.08*** | 0.37*** | 1.00 |  |  |  |  |  |  |  |
| 12. Hyperactivity | 0.29*** | 0.46*** | 0.68*** | 0.30*** | 0.10*** | 0.05*** | -0.11*** | -0.12*** | 0.15*** | 0.35*** | 0.55*** | 1.00 |  |  |  |  |  |  |
| 13. Peer problems | 0.37*** | 0.31*** | 0.33*** | 0.56*** | 0.03** | 0.07*** | -0.06*** | -0.08*** | 0.04*** | 0.49*** | 0.37*** | 0.34*** | 1.00 |  |  |  |  |  |
| 14. Delay aversion | 0.05*** | 0.09*** | 0.10*** | 0.05*** | 0.16*** | -0.01 | -0.05*** | -0.07*** | 0.09*** | 0.04*** | 0.11*** | 0.10*** | 0.04*** | 1.00 |  |  |  |  |
| 15. Deliberation time | 0.06*** | 0.07*** | 0.12*** | 0.09*** | 0.008 | 0.31*** | -0.18*** | -0.09*** | 0.02 | 0.08*** | 0.07*** | 0.12*** | 0.12*** | -0.12*** | 1.00 |  |  |  |
| 16. Quality of decision-making | -0.07*** | -0.09*** | -0.13*** | -0.08*** | -0.06*** | -0.08*** | 0.34*** | 0.15*** | -0.008 | -0.08*** | -0.11*** | -0.14*** | -0.08*** | -0.05*** | -0.37*** | 1.00 |  |  |
| 17. Risk adjustment | -0.08*** | -0.11*** | -0.13*** | -0.09*** | -0.04*** | -0.08*** | 0.23*** | 0.25*** | -0.02* | -0.10*** | -0.14*** | -0.14*** | -0.09*** | -0.18*** | -0.19*** | 0.36*** | 1.00 |  |
| 18. Risk-taking | -0.02* | 0.08*** | 0.09*** | 0.005 | 0.07*** | -0.04*** | 0.03* | -0.04*** | 0.33*** | -0.04*** | 0.07*** | 0.12*** | 0.001 | 0.21*** | -0.06*** | 0.08*** | -0.21*** | 1.00 |

* *p* < 0.05; ** *p* < 0.01; *** *p* < 0.001

r = 0.00 to 0.29 = small coefficients; r = 0.30 to 0.49 medium coefficients; r = 0.50 to 1.00 large coefficients.

SDQ Strengths and Difficulties Questionnaire. CGT Cambridge Gambling Task.

Table S4 – Model A: Results of unadjusted cross-lagged models examining the relationship between SDQ and CGT at age 11 with SDQ and CGT at age 14 (outcomes)

|  | **SDQ age 14** | | | |  | **CGT age 14** | | | | |
| --- | --- | --- | --- | --- | --- | --- | --- | --- | --- | --- |
|  | *Emotional problems* | *Conduct problems* | *Hyperactivity* | *Peer problems* |  | *Delay aversion* | *Deliberation time* | *Quality of decision-making* | *Risk adjustment* | *Risk-taking* |
|  | *b* (SE) | *b* (SE) | *b* (SE) | *b* (SE) |  | *b* (SE) | *b* (SE) | *b* (SE) | *b* (SE) | *b* (SE) |
| **SDQ age 11** |  |  |  |  |  |  |  |  |  |  |
| *Emotional problems* | 0.56*** (0.01) | – | – | – |  | -0.00006 (0.002) | -1.32 (7.84) | 0.0003 (0.001) | -0.008 (0.007) | -0.004*** (0.001) |
| *Conduct problems* | – | 0.64*** (0.02) | – | – |  | 0.006** (0.002) | 6.21 (10.15) | -0.002 (0.001) | -0.03*** (0.009) | 0.006*** (0.001) |
| *Hyperactivity* | – | – | 0.66*** (0.01) | – |  | 0.005*** (0.001) | 35.73*** (6.21) | **-0.004*** (0.001)** | -0.03*** (0.006) | **0.003*** (0.001)** |
| *Peer problems* | – | – | – | 0.60*** (0.01) |  | 0.002 (0.002) | **23.82** (9.07)** | -0.0009 (0.001) | -0.01 (0.008) | -0.002 (0.001) |
| **CGT age 11** |  |  |  |  |  |  |  |  |  |  |
| *Delay aversion* | -0.02 (0.12) | 0.18 (0.09) | 0.09 (0.10) | 0.04 (0.09) |  | 0.14*** (0.01) | – | – | – | – |
| *Deliberation time* | 0.00003 (0.00002) | 8.48e-06 (0.00001) | 6.62e-06 (0.00002) | **0.00004* (0.00002)** |  | – | 0.24*** (0.02) | – | – | – |
| *Quality of decision-making* | -0.05 (0.16) | -0.24 (0.12) | **-0.58*** (0.14)** | -0.16 (0.12) |  | – | – | 0.27*** (0.01) | – | – |
| *Risk adjustment* | -0.07* (0.03) | -0.03 (0.02) | -0.03 (0.02) | -0.06** (0.02) |  | – | – | – | 0.23*** (0.01) | – |
| *Risk-taking* | -0.26 (0.17) | 0.04 (0.12) | **0.51*** (0.15)** | -0.007 (0.12) |  | – | – | – | – | 0.28*** (0.01) |

* *p* < 0.05; ** *p* < 0.01; *** *p* < 0.001

**Bold** = bidirectional relationships.

SDQ Strengths and Difficulties Questionnaire. CGT Cambridge Gambling Task. *b* coefficient. SE standard error.

Model adjusted for autoregressive paths of SDQ and CGT measures and for the confounders, i.e. child’s gender, ethnicity, pubertal status, and cognitive ability, and maternal education.

Note: The coefficients should be interpreted in the context of and according to the scale of measurement of the variables.

Table S5 – Model B: Results (standardised regression coefficients and their standard errors) of adjusted cross-lagged models examining the relationship between SDQ and CGT at age 11 with SDQ and CGT at age 14 (outcomes)

|  | **SDQ age 14** | | | |  | **CGT age 14** | | | | |
| --- | --- | --- | --- | --- | --- | --- | --- | --- | --- | --- |
|  | *Emotional problems* | *Conduct problems* | *Hyperactivity* | *Peer problems* |  | *Delay aversion* | *Deliberation time* | *Quality of decision-making* | *Risk adjustment* | *Risk-taking* |
|  | *β* (SE) | *β* (SE) | *β* (SE) | *β* (SE) |  | *β* (SE) | *β* (SE) | *β* (SE) | *β* (SE) | *β* (SE) |
| **SDQ age 11** |  |  |  |  |  |  |  |  |  |  |
| *Emotional problems* | 0.51*** (0.01) | – | – | – |  | -0.003 (0.01) | -0.003 (0.02) | 0.008 (0.02) | 0.001 (0.02) | -0.04** (0.01) |
| *Conduct problems* | – | 0.61*** (0.02) | – | – |  | **0.04** (0.02)** | 0.004 (0.02) | -0.01 (0.02) | -0.04** (0.01) | 0.06*** (0.01) |
| *Hyperactivity* | – | – | 0.66*** (0.01) | – |  | 0.05** (0.02) | 0.07*** (0.02) | **-0.06** (0.02)** | -0.06*** (0.02) | 0.02 (0.02) |
| *Peer problems* | – | – | – | 0.55*** (0.01) |  | 0.02 (0.02) | **0.03* (0.02)** | -0.002 (0.02) | -0.01 (0.01) | -0.03* (0.01) |
| **CGT age 11** |  |  |  |  |  |  |  |  |  |  |
| *Delay aversion* | 0.004 (0.01) | **0.03* (0.01)** | 0.006 (0.01) | 0.002 (0.01) |  | 0.16*** (0.01) | – | – | – | – |
| *Deliberation time* | 0.01 (0.01) | 0.003 (0.01) | 0.003 (0.01) | **0.03* (0.01)** |  | – | 0.33*** (0.02) | – | – | – |
| *Quality of decision-making* | -0.004 (0.01) | -0.02 (0.01) | **-0.03** (0.01)** | -0.006 (0.01) |  | – | – | 0.33*** (0.01) | – | – |
| *Risk adjustment* | -0.01 (0.01) | -0.01 (0.01) | -0.01 (0.01) | -0.03* (0.01) |  | – | – | – | 0.22*** (0.01) | – |
| *Risk-taking* | 0.008 (0.01) | 0.003 (0.02) | 0.02 (0.01) | -0.01 (0.01) |  | – | – | – | – | 0.27*** (0.01) |

* *p* < 0.05; ** *p* < 0.01; *** *p* < 0.001

**Bold** = bidirectional relationships.

SDQ Strengths and Difficulties Questionnaire. CGT Cambridge Gambling Task. *β* standardised coefficient. SE standard error.

Model adjusted for autoregressive paths of SDQ and CGT measures and for the confounders, i.e. child’s gender, ethnicity, pubertal status, and cognitive ability, and maternal education.

Note: The coefficients should be interpreted in the context of and according to the scale of measurement of the variables.

MeSH Terms used in literature search in PubMed

**Mental health**

- Affective Symptoms
- Psychological Distress
- Depression
- Anxiety
- Peer Group
- Behavioral Symptoms
- Conduct Disorder
- Disruptive, Impulse Control, and Conduct Disorders
- Antisocial Personality Disorder
- Attention Deficit Disorder with Hyperactivity
- Mental Health

**Affective decision-making & Gambling tasks**

- Decision Making
- Delay Discounting
- Risk Taking
- Reward
- Punishment

**Population**

- Child
- Adolescent
